# Building an international network for a primary care research program: reflections on challenges and solutions in the set-up and delivery of a prospective observational study of acute cough in 13 European countries

**DOI:** 10.1186/1471-2296-12-78

**Published:** 2011-07-27

**Authors:** Jacqueline Nuttall, Kerenza Hood, Theo JM Verheij, Paul Little, Curt Brugman, Robert ER Veen, Herman Goossens, Christopher C Butler

**Affiliations:** 1 South East Wales Trials Unit (SEWTU), Department of Primary Care and Public Health, School of Medicine, Cardiff University, Neuadd Meirionnydd, Heath Park, Cardiff, UK; 2 University Medical Center Utrecht, Julius Center for Health, Sciences and Primary Care, Universiteitsweg 100, Stratenum, 6th Floor, 6.111, 3584 CX Utrecht, The Netherlands; 3 University of Southampton, Southampton, SO16 5ST, UK; 4 Campus Drie Eiken, D.S313, Universiteitsplein 1, 2610 Wilrijk, Belgium

## Abstract

**Background:**

Implementing a primary care clinical research study in several countries can make it possible to recruit sufficient patients in a short period of time that allows important clinical questions to be answered. Large multi-country studies in primary care are unusual and are typically associated with challenges requiring innovative solutions. We conducted a multi-country study and through this paper, we share reflections on the challenges we faced and some of the solutions we developed with a special focus on the study set up, structure and development of Primary Care Networks (PCNs).

**Method:**

GRACE-01 was a multi-European country, investigator-driven prospective observational study implemented by 14 Primary Care Networks (PCNs) within 13 European Countries. General Practitioners (GPs) recruited consecutive patients with an acute cough. GPs completed a case report form (CRF) and the patient completed a daily symptom diary. After study completion, the coordinating team discussed the phases of the study and identified challenges and solutions that they considered might be interesting and helpful to researchers setting up a comparable study.

**Results:**

The main challenges fell within three domains as follows:

i) selecting, setting up and maintaining PCNs;

ii) designing local context-appropriate data collection tools and efficient data management systems; and

iii) gaining commitment and trust from all involved and maintaining enthusiasm.

The main solutions for each domain were:

i) appointing key individuals (National Network Facilitator and Coordinator) with clearly defined tasks, involving PCNs early in the development of study materials and procedures.

ii) rigorous back translations of all study materials and the use of information systems to closely monitor each PCNs progress;

iii) providing strong central leadership with high level commitment to the value of the study, frequent multi-method communication, establishing a coherent ethos, celebrating achievements, incorporating social events and prizes within meetings, and providing a framework for exploitation of local data.

**Conclusions:**

Many challenges associated with multi-country primary care research can be overcome by engendering strong, effective communication, commitment and involvement of all local researchers. The practical solutions identified and the lessons learned in implementing the GRACE-01 study may assist in establishing other international primary care clinical research platforms.

**Trial registration:**

ClinicalTrials.gov Identifier: NCT00353951

## Background

Implementing primary care research in several countries carries many benefits. It eliminates the wasteful duplication of research efforts, brings together many experts in one field and increases the likelihood of successful recruitment within a shorter period of time. Given that large sample sizes are needed to answer some major research questions with properly powered planned sub-group analyses, such multi-country studies are becoming more common. Inter-country comparisons can also help to determine the influence of contextual, health service and cultural variations across clinical outcomes. Implementing such studies, however, is difficult. Regulatory and ethical principles in implementing clinical trials and other relevant empirical clinical research designs are well documented in the International Committee on Harmonisation Good Clinical Practice (ICH-GCP) guidelines and each national regulatory document. The European Clinical Research Infrastructure Network (ECRIN), funded by the 6th Framework Program of the European Commission, aims to bridge the fragmented organization of European clinical research and support EU-wide clinical research. However, the few accounts of practical operational aspects, challenges faced and solutions developed when setting up multi-country studies have hitherto either been diseased focussed [1,2] or study design specific [3]. International studies require some consideration and negotiation of language and cultural barriers, the remote nature of site set-up and coordination, differences in ethical issues and approval processes and variations in openness to new ways of doing things [4].

**Figure 1:**
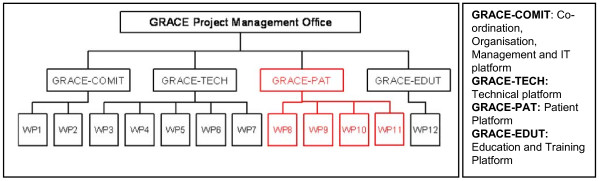
**An overview of the GRACE platforms and work-packages**.

Genomics to combat Resistance Against antibiotics in Community acquired LRTI in Europe (GRACE) Network of Excellence, was funded by the European Union and consisted of four work platforms and 12 work packages (See Figure 1 for an overview of the GRACE platforms and work packages. - The patient platform is highlighted in red; see http://www.grace-lrti.org for more details). One of the main aims of GRACE was to establish a multi-disciplinary network of research to address a complex problem and to establish an enduring European-wide primary care research network for future research. The first GRACE clinical study, GRACE-01, was a multi-European investigator-driven prospective, observational study which aimed to set up 14 Primary Care Networks (PCN) to support the GRACE patient clinical research platform to develop and implement GRACE clinical studies, and to describe the presentation, management and outcome of acute cough in primary care in contrasting European countries.

Our experiences in the GRACE 01 study may be helpful to others setting up European research studies in primary care.

## Methods

This paper does not present the findings or full methods of the GRACE 01 Study which are reported elsewhere,[5]. Rather it presents some reflections over key challenges faced and the solutions devised when setting up an international European primary care network and when implementing a large, international clinical study. Comprehensive notes were kept by the GRACE 01 study manager (JN) and the work package leader (CCB), and discussions and meetings were held with other researchers in the site coordinating GRACE 01 (KH), those responsible for producing the GRACE On-line data System (GOS) (CB and RV), other work package leaders who were part of the clinical platform (TVH and PL), and the co-ordinator of the Grace Network of Excellence (HG). JN and CCB drafted an outline of major lessons learned and all other authors commented on, and modified the draft through several iterations. Some details of GRACE and the research methods of GRACE 01 are provided below to indicate the complexity and challenges of the study.

### Over view of study methods and PCNs

**Figure 2:**
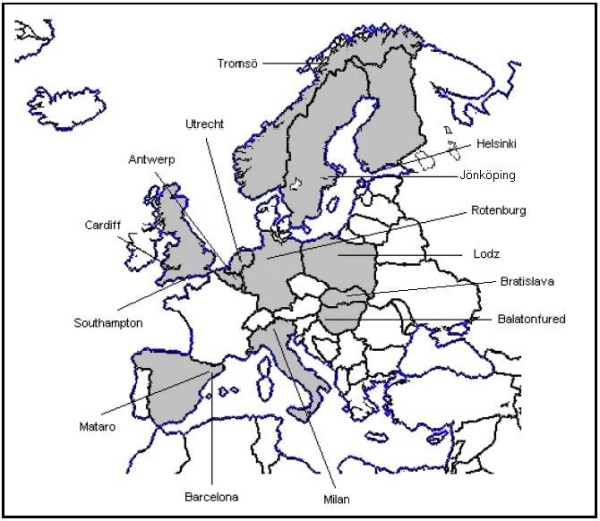
**Map of Primary Care Networks Participating in GRACE-01**.

GRACE-01 was a prospective observational study based on a study population of Clinicians from 14 primary care research networks in 13 European countries (see Figure 2 for GRACE map). Clinicians were asked to enrol consecutive adult patients with an illness where an acute or worsened cough was the main or dominant symptom, or where a clinical presentation that suggested a lower respiratory tract infection, with duration of up to and including 28 days was observed. We aimed to enrol a minimum of 300 patients per PCN and between 3600 and 4000 patients overall.

### Study materials and procedures

GRACE-01 used two surveys in the format of both a Case Report Form (CRF) that was completed by the Clinician in the initial consultation and a 28-day symptom diary that was completed by the patient. The topics covered in the CRF and diary are listed in Table 1. The study documents required by ethics review committees and participants (Clinicians and patients) were translated from English into local languages. "Back translation" in to English were carried out.

**Table 1:** Areas covered in the CRF and the patient symptom diary

CRF question areas	Patient Diary question areas
Location of the consultation	Main reasons for consulting

14 Symptoms and their reported severity at presentation	Daily rating of 13 symptoms

Co-morbidity	Questions about the present illness

Temperature	Smoking history

Physical examinations performed and findings	Social demographic factors (e.g. educational qualifications, job, numbers of persons living in the house)

Investigations ordered (e.g. blood tests and × rays)	Use of health care facilities - visited and contacted (e.g. GP, Nurse, pharmacist)

Referral	Recovery

Treatment details including antibiotics and over the counter medications	Expectations about treatment

Follow up arrangements	Hospital admission

Advice about work	Weekly questions ask about medication use, work attendance, brief quality of life questions (EQ-5D).

Patient expectations	Beliefs about antibiotics.

Perceptions of patient satisfaction	

### Data management

A web based platform was developed (Research Online) that served the following purposes:

• to function as a central communication medium comprising a web site and a library for study related documents,

• to ensure that online data capturing and data validation was compliant with regulatory requirements,

• to support different languages,

• to provide a central storage resource in an encrypted database, and

• to provide real time monitoring of the progress of the study throughout all participating PCNs.

This platform was referred to as the 'GRACE-Online-System' (GOS). Either Clinicians or NNFs entered the study data. Each user (Clinician and NNF) accessed the system via a personal password protected account.

### Recruitment

Participating Clinicians were asked to recruit consecutive eligible patients from October to November 2006, and from late January to March 2007.

### The GRACE structure

**Figure 3:**
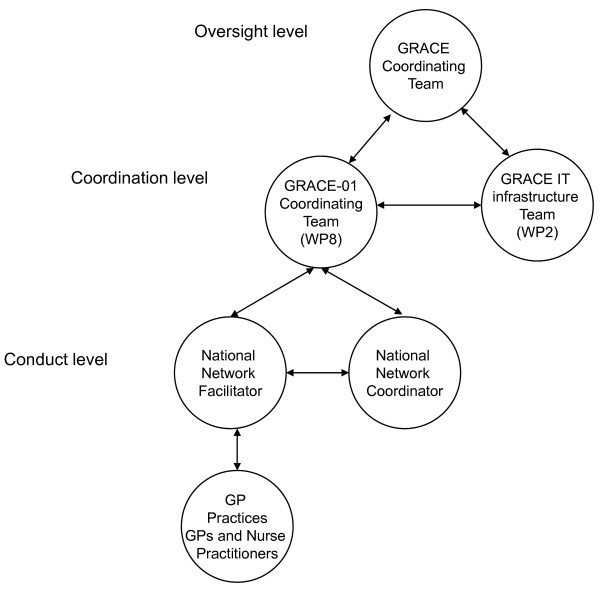
**Organizational structure of GRACE-01**.

GRACE had a central coordinating team at the University of Antwerp who took overall responsibility for all GRACE work packages. Each work package had its own lead and coordinating team to manage the studies or tasks contained in their work package (for example the GRACE-01 coordinating team was located in Cardiff and the GRACE IT infrastructure team was located in Utrecht). The Study Manager within the GRACE-01 organising team set up and managed the study and PCNs. Each PCN had a National Network Coordinator (NNC) and National Network Facilitators (NNF) who coordinated the study locally. Figure 3 shows the organisational structure of GRACE-01.

### PCN set up

Some 14 PCNs with a research interest in respiratory conditions were recruited during the winter of 2005. An NNC and an NNF were appointed to act as points of communication with the coordinating team and to cascade information to local sites. These individuals were also given responsibility for implementing the study. At least one of these appointees was clinically qualified. The main responsibilities of the NNCs were to administer finance arrangements and contractual obligations within the PCN, ensure effective communication with local investigators and with the coordinating team, to organise translations and back translation of study materials and to develop awareness of GRACE throughout the network. NNCs were also tasked with ensuring recruitment targets were met and study procedures were carried out according to Good Clinical Practice (GCP) and local regulatory requirements. The main responsibilities of the NNFs were to gain ethical approval, to cascade training to Clinicians, to ensure written informed consent was obtained from all participating clinicians and patients, and to enter data in a timely manner on to the GRACE-Online-System. NNFs were also tasked with scheduling reminder phone calls to participants to request the return of diaries. They were also asked to collect missing data, to ensure patient data were properly stored, and to liaise with the coordinating team regarding data cleaning.

Subcontract agreements were signed between the PCNs and the sponsor of GRACE-01; Cardiff University. A template GP agreement was designed for PCNs for use with their Clinicians.

All NNCs and NNFs participated in an intensive one-day training session that included interactive online data entry system demonstrations and exercises, a patient recruitment simulation using a study pack detailing how to cascade GCP and other study-relevant training to recruiting clinicians. Training sessions also included information about data protection, and role descriptions communicated to participants.

During the set up and study implementation phase, NNCs and NNFs were able to email or telephone the GRACE 01 study manager about any concerns, who responded in almost all instances on the same day.

We planned to separate out the recruitment periods to incorporate a period of reflection. A second recruitment drive was undertaken two months after the initial recruitment exercise and each element (functioning of PCN, recruitment, data collection and management and study processes) of GRACE-01 was assessed in detail. We also held a face-to-face meeting with NNFs/NNCs to share good practice and to problem-solve ahead of the second recruitment period. This enabled us not only to assess how to improve recruitment and follow up, but also to evaluate and reflect on challenges that had arisen and solutions developed. At the end of the study, the patient platform work package team leads, NNFs and NNCs reflected in an annual GRACE meeting over lessons learned from conducting this European Study.

## Results

We considered that the main challenges of GRACE-01 fell within three domains as follows;

i) selecting, setting up and maintaining PCNs;

ii) designing local context-appropriate data collection tools and efficient data management systems; and

iii) gaining commitment and trust from all involved whilst maintaining enthusiasm.

(See additional file 1: Table detailing the challenges and solutions)

### Selecting, setting up and maintaining PCNs

The initial challenge was to recruit 14 sufficiently large, PCNs from contrasting countries. Pre-defined, selection criteria were established; specifically, patient population covered (access to at least 20,000 enlisted patients), their ability to carry out GRACE tasks, their anticipated budgets for carrying out GRACE-01 and finally, geographical spread (we wanted selected PCNs to include northern, southern eastern and western Europe to allow comparisons between contrasting health systems within the study). Contacts established through annual meetings of an international network of researchers with a special interest in the disease topic (The General Practice Respiratory Infections Network) were invaluable in this process. Each interested PCN completed a questionnaire detailing their ability to meet the selection criteria and the work package leads in the Clinical Platform together with the overall project coordinator made the final selection.

We found that selecting NNCs and NNFs who had a prior interest in infections and who were committed to the wider mission of the GRACE Network of Excellence led to successful study implementation at each respective site. It became clear that roles and responsibilities and communication strategies were key to eradicating uncertainty and poor performance of both individual NNF/NNC and PCN as a whole. Comprehensive and systematic roles and responsibilities of the PCNs were therefore discussed and agreed with the NNC and NNF. A description of the importance and procedures for disseminating GRACE-01 information to network clinicians was essential in ensuring effective communication and enthusiasm. All NNCs and NNFs were appraised of the study design and set up issues through face-to-face meetings, teleconferences and e-mail. Their contributions to modifying and finalising these instilled a sense of ownership within each PCN.

PCNs considered the face-to-face training to be especially constructive. We found that within a multi lingual environment, interactive sessions with discussion prompts were useful in ensuring key information items were understood. A lunchtime social activity (a visit to a museum) allowed time to chat and refresh minds ahead of the afternoon sessions. This one day training session provided the opportunity to meet with colleagues from other PCNs and to share anxieties about implementation and possible solutions. The session resulted in a shared sense of camaraderie.

Clear, achievable Standard Operating Procedures (SOPs) and feasible specific working practices were developed by the GRACE -01 coordinating team to support the NNCs and NNFs in their every day roles. We provided PCNs with SOPs for all generic procedures (such as data entry instructions, PCN file contents and maintenance, contacting patients via telephone, data querying, database lock down and study closure *inter alia*) to ensure consistency across PCNs. Where local flexibility was permissible, this was made clear, and certain procedures were devised for PCNs to amend and adapt to suit their individual context. However, PCNs were required to log adaptations to procedures where appropriate.

The gap between data collection periods proved useful. Based on experiences from the first phase, we made slight changes to the data collection tools and online system towards a more user-friendly interface. Examples of this included making illustrative questions clearer, emphasising where only one box should be ticked (rather than "multi-punch") and including a "not applicable" option where appropriate. We re-trained individuals where skills development requirements were observed through monitoring reports. During the face-to-face reflection meeting, initiatives were suggested by both the coordinating team and the NNCs and NNFs to maximize future recruitment and patient diary response rates. These included re-training clinicians in taking consent, involving practice receptionists in recruitment, telephoning patients in the evening for follow up, sending letters to patients when they were not contactable by telephone, and using a simple flow diagram for patients detailing processes for completing the dairy (a complete list is presented as Table 2). In this way, over 3,400 patients were recruited overall. Five of the networks each recruited 300 or more patients. The overall CRF return rate was 99%, and the patient diary return rate was 80%. Four PCNs received a response rate of over 90% of patient diaries.

**Table 2:** Suggestions for improving recruitment and patient responses

Improve Recruitment	Improve patient Diary Response Rate
Appoint key individuals within surgeries/health centers responsible for the running of GRACE-01.	Send a letter when the patient is unable to be contacted by phone between days 4-7

Practice receptionists give patient information sheet to all patients attending with a cough to read in the waiting room; incentives for receptionist (e.g. £5 voucher per patient recruited).	Clinicians register if the patient has a preferred contact telephone number (e.g. mobile phone) and the best time of day to contact them.

Clinicians to reserve slots in their routine consultation schedules, clearly identified as GRACE-01 slots. OR Ask clinicians to plan ahead slots in their consultation schedule with the subject GRACE-01. This will also remind them of the study	Flow diagram at the beginning of the patient diary detailing process for completing the patient diary. Use different colored paper for general questions that are separate from the daily questions to help ensure patients do not miss questions.

Increase clinician involvement by giving them a GRACE certificate of participation.	Advertise and hold a lottery with all patients who have returned a patient diary as an incentive to complete and return the patient diary

Refresher/re-training clinicians in the consent process of GRACE-01 procedures	Shortened version of patient diary with selected key main outcome questions when no patient diary has been returned.

### Designing local context-appropriate data collection tools and efficient data management systems

Specific challenges in designing data collection tools for multiple countries include language and translation issues, differences in cultural norms and perceptions, differences in healthcare structure, and ethical issues. A complex, bespoke data management system was required to accommodate this variation.

We involved representatives from all of the PCNs in designing the CRFs/Diary. Detailed discussions were necessary to agree and formulate questionnaire items that could be translated to achieve consistent meaning across all participating countries. Each of the NNCs and NNFs were asked to comment on all drafts of the CRFs and patient diaries. Relevant cultural, healthcare structure or cost differences were documented and, where relevant, were excluded or amended from country specific CRFs/Diaries.

Although some PCNs felt that completing a CRF in English might be acceptable for clinicians, we nevertheless translated all documents into local languages (including clinician orientated materials) as we believed this would increase completion rates by engendering a greater sense of local ownership. In most cases, documents were translated by the NNC, ensuring consistency and accuracy as a result of their involvement in the design of surveys. Quality assurance was achieved by "back translations" whereby someone other than the NNC (who was proficient in both the local language and English) translated the local language version back into English without sight of the original English version. Back translations were checked against the original English versions by the GRACE-01 coordinating team, and inconsistencies were addressed in discussions with all NNCs. Clinical input in this process was particularly helpful in ensuring consistency of terms used to describe clinical signs.

Data entry and management was facilitated via the GRACE-Online-System (GOS), which also allowed the coordinating team to monitor data completeness and data quality. Reports identified values that lay outside pre-definite ranges. Clinicians were offered two data entry options, either directly onto GOS, or first by completing a paper based version that was entered onto GOS at a later point by the NNF. The GOS platform tracked data entry to individuals and logged the time lag between initial collection and data entry. This provided the opportunity to rapidly address the timeliness of data entry with responsible individuals. In general, recruiting clinicians preferred not to complete the CRF online in front of their recruited patients, as they were cautious of this impacting negatively on doctor-patient interaction.

Real time reports were developed for the NNF to monitor inclusion rates for each clinician. A so-called "workflow" was implemented to indicate to the NNF and recruiting clinicians which surveys needed to be filled out at what time, as well as to remind them of outstanding tasks that needed to be carried out for each individual patient. GOS generated a list of tasks which remained visible on the system until they were completed. This online data entry system ensured completeness and quality of data and reduced the burden on the GRACE-01 coordinating team.

Close monitoring by the GRACE coordinating team, in real time, was pivotal to ensure standards remained consistent. Reports on patient recruitment rates, CRF completion rates, patient diary return rates, and the timeliness of data entry were assessed weekly for each PCN by the GRACE 01 Study Manager. These reports identified instances where more help and encouragement for PCNs was required. The reports also generated data for the weekly GRACE-01 newsletters, which reported study progress.

We asked all NNFs to translate or categorize free-text entries at the point of data entry.

### Gaining commitment and trust and maintaining enthusiasm

A shared sense of the importance of the research questions to improving clinical care was the foundation for establishing a common purpose and a spirit of camaraderie. Strong leadership and frequent communication meant that NNCs and NNFs got to know well and grew to trust the GRACE-01 coordinating team. NNCs and NNFs were involved in the early development of the study and were included in meetings where they could contribute effectively to the study question and where they were invited to comment on materials and the study process. This ensured that the NNCs and NNFs felt scientifically responsible and accountable for the integrity of the study [6]. A social run or walk, called the "Race for GRACE" at a later annual study meeting, facilitated relationship building and relaxation. PCN members regularly submitted news articles for the quarterly newsletter. Prizes were regularly awarded for the fastest recruiting PCN, and photos were taken and distributed. PCN members regularly contributed items to the quarterly GRACE newsletters. A coherent GRACE ethos came to be established and partners and collaborators informally referred to themselves as the "GRACE Family".

Communication between NNCs, NNFs and the GRACE-01 coordinating team were encouraged. They were asked to get in touch immediately if anything was not clear of if they had identified a problem. Regular meetings and teleconferences, usually weekly, were set and an inviting open and friendly environment was created. At each of these, participants were specifically invited to contribute reflections and suggestions, and identify problems. An email distribution list allowed answers to queries and comments from individuals to be shared with all NNCs and NNFs, providing them with a single port of call for advice and to share relevant information. Communication was maintained between the GRACE 01 Study Manager and PCNs from day to day. All queries were acknowledged and most were answered within 24 hours. Lessons were shared across all PCNs quickly and effectively. GRACE-01 Newsletters and recruitment updates were sent out weekly. These were designed to encourage all PCNs responsible for areas such as patient recruitment, data entry or CRF/Dairy return rates. A competitive spirit was fostered by presenting histograms that allowed PCNs to compare their performance with other PCNs. Frequently Asked Questions (FAQs) and answers were entered on the GOS for all to access. Annual face-to-face meetings reinforced commitment to specific studies and the overall mission of GRACE encouraged discussions around problems and locally developed solutions.

A publication policy was developed detailing criteria for authorship and a list of planned GRACE-01 publications. PCN members were given the opportunity to state which of the planned GRACE-01 papers they would like to contribute towards in terms of authorship. Exploitation of local data PCN was also encouraged by asking PCNs to suggest their own country-specific research questions. This provided the opportunity for many within the networks to give presentations and write journal articles about their country specific results and to feedback emerging data to network members.

## Discussion

To our knowledge, this is the first example of a multi- national primary care research network implementing a study in the field of common infections of this size and complexity in Europe. The key challenges we describe were setting up and maintaining the PCNs, ensuring that the data collection tool and data management instructions were culturally applicable, and maintaining communication and enthusiasm. The first clinical study of the GRACE Network of Excellence was implemented over a short time scale and to a high standard. Key factors in achieving this were:

• selecting, setting up and appointing key individuals (NNFs and NNCs) with clearly defined roles in each PCN

• involving PCNs early in the development of the study materials and research procedures

• designing local context-appropriate data collection tools and efficient data management systems

• scrupulously checking back translations to accurately reflect the originals, ensuring that even small differences in wording were identified.

• closely monitoring each PCN's progress using the centralised data collection system to generate reports of accrual related data and outstanding tasks.

• setting out clear, well publicised standard operating procedures

• gaining commitment and trust from all involved and maintaining enthusiasm.

• providing strong leadership, encouraging communication between study coordinators and all key PCN personnel

• establishing high level agreement about the value of the research and a shared common sense of purpose 

• recognising achievements of PCNs and incorporating social events and prizes for the PCNs during large annual study meetings.

• providing a framework for, and encouraging contributions towards publications including earning authorship and encouraging additional exploitation of local data

We recommend that researchers should not underestimate the level of communication required to ensure a successful study of this nature. Appropriate resources should be identified for face-to-face meetings/teleconferences and annual events, since getting to know fellow researchers within a network helps to attract commitment and a sense of common purpose and camaraderie. An interactive (rather than didactic) approach to training a multilingual group worked well.

There are certain things we could have done better;

For example, we relied upon our NNCs to translate the study documents into local languages and then, most commonly, it was a colleague who "back translated" the translation. One of the GRACE-01 coordinating team then checked the back translation against the original English document. This worked well. However, this process may still result in an inappropriate translation, particularly of technical terms. Multiple forward translations and comparing the results of each is another possible approach [7,8].

Separate ethical and other regulatory approvals were required for all PCNs. The time taken to obtain this approval, however, varied considerably between PCNs for different reasons including the frequency of ethical review board meetings which ranged from every week with 28 day approval time (Utrecht and Antwerp) to every month with 60 days approval time (UK-Cardiff and Southampton, and Balatonfüred). Ethics review board meetings required different levels of information (for example on data protection, record keeping, insurance coverage information and so forth), which impacted on the time it took to gain ethical approval. In hindsight, although all PCNs opened to recruitment on time, a more thorough knowledge of local differences would have been helpful to establish more easily manageable time lines for gaining ethical approval. However, with the development of ECRIN, this will potentially be easier for future studies. In general, we underestimated the time it took to gain ethical, Research and Development and other regulatory approvals.

In addition, free text fields were observed to be more time consuming and laborious to 'clean' and analyze. Although we established procedures to manage this, we underestimated the time taken and complexity of managing free text fields. We would, in future, attempt to keep fixed category responses to an absolute minimum and to limit the number of "other" options available.

The follow up of patients ranged from 60% to 100% between PCNs. Approaches to enhancing accrual in one PCN did not necessarily work well in another. A more flexible, locally tailored patient follow up plan might have improved the follow up rates within the PCNs where follow up rates were low.

### Comparison to other research

An account of operational challenges in implementing large clinical trials in a resource poor setting [9] identified many similar challenges and solutions to successful coordination and to the importance of establishing clear SOPs. Also important here are the monitoring systems adhered to and creating detailed role and responsibility descriptions which are identified as key to successful research implementation.

### Potential strengths and weaknesses

Recruited PCNs are likely to over-represent research-interested health care professionals and many of these will be affiliated to universities. However, in most countries, we were able to include full-time service practices, many of which participated in research for the first time. The organization of primary health care and available human resources facilitated the research in some countries more than others. The commitment of participating clinicians seemed to have a greater bearing on successful patient recruitment than available resources. The Belgium PCN, for instance, was considered one of the best recruiting networks despite primary care practices being mostly single-handed and where research nurses were unavailable. Excellent recruitment in Belgium was achieved because the study team were motivated and well supported.

## Conclusion

We aimed to set up a European-wide primary care research network to deliver an ambitious observational study during one winter period. We succeeded in establishing a clinical platform for the GRACE 01 study, and many of the PCNs have continued to recruit patients into subsequent GRACE studies. We achieved recruitment targets in many PCNs in GRACE 01. GRACE 01 continues to generate data that has clinical relevance [10-12].

Despite the complexity of conducting this international study in primary care, strong communication and commitment across all local NNFs/NNCs meat that it was possible to set up an effective international, multi-centre primary care research network. Recruiting research participants continues to be a challenge however [13-15]. This description of the challenges and some of the solutions we implemented may assist others in establishing effective, enduring international clinical research platforms in primary care.

## Competing interests

The authors declare that they have no competing interests.

## Authors' contributions

All authors contributed to either the conception and design, or the analysis and interpretation of the data. All authors contributed to drafting and revising the manuscript. All authors have approved this final version of the manuscript. No one else who fulfils these criteria has been excluded.

## Pre-publication history

The pre-publication history for this paper can be accessed here:

http://www.biomedcentral.com/1471-2296/12/78/prepub

## Supplementary Material

Additional file 1 **Table 3: Challenges and solutions in tabulated format - this table details all the challenges and solutions observed in the GRACE-01 Study**. Click here for file

## References

[B1] Knot J W P Wijns W Stenestrand U Kristensen SD Van' T Hof A Weidinger F Janzon M Nörgaard BL Soerensen JT van de Wetering H Thygesen K Bergsten PA Digerfeldt C Potgieter A Tomer N Fajadet J How to set up an effective national primary angioplasty network: lessons learned from five European countries EuroIntervention 2009 3 5 299 301-309 19736153

[B2] Mathai J B A Cranswick N Lessons learnt in conducting a clinical drug trial in children with Asperger Syndrome Australasian Psychiatry 2005 13 2 173 175 15948916 10.1080/j.1440-1665.2005.02183.x

[B3] Bonnefoy X B M Davidson M Robbel N A pan-European housing and health survey: description and evaluation of methods and approaches International Journal of Environment and Pollution 2007 30 3/4 363 383 10.1504/IJEP.2007.014816

[B4] Bosch X T S Cultural challenges and international research integrity Lancet 2009 373 9664 610 612 10.1016/S0140-6736(09)60379-2 19231616

[B5] Butler CC H K Verheij T Little P Melbye H Nuttall J Kelly MJ Molstad S Godycki-Cwirko M Almirall J Torres A Gillespie D Rautakorpi U Coenen S Goossens H Variation in antibiotic prescribing and its impact on recovery in patients with acute cough in primary care: prospective study in 13 countries BMJ 2009 338 jun23_2 b2242 19549995 10.1136/bmj.b2242 PMC3272656

[B6] Bangdiwala SI DP C Ramiro LS Munroz SR Coordination of international multicenter studies: governance and administrative structure Salud pública Méx [online] 2003 45 1 58 66 12649963 10.1590/s0036-36342003000100008

[B7] Maxwell B MMaK DL Translation and Cultural Adaptation of the Survey Instruments, in Third International Mathematics and Science Study (TIMSS) Technical Report; Design and Development 1996 Chestnut Hill

[B8] Ervin S B R Translation Problems in International Surveys Public Opin Q 1952 16 4 595 604 10.1086/266421

[B9] Cutts FT E G Zaman SMA Yallop FG Operational Challenges in Large Clinical Trials: Examples and Lessons Learned from the Gambia Pneumococcal Vaccine Trial PLOS Clin Trial 2006 1 3 e16 10.1371/journal.pctr.0010016 PMC1500815 16871317

[B10] Butler CC Treatment of acute cough/lower respiratory tract infection by antibiotic class and associated outcomes: a 13 European country observational study in primary care The Journal of antimicrobial chemotherapy 2010 65 11 2472 8 10.1093/jac/dkq336 20852271

[B11] Wood J Antibiotic prescribing for adults with acute cough/LRTI: congruence with guidelines The European respiratory journal: official journal of the European Society for Clinical Respiratory Physiology 2011

[B12] Butler CC Antibiotic prescribing for discoloured sputum in acute cough/LRTI The European respiratory journal: official journal of the European Society for Clinical Respiratory Physiology 2011 10.1183/09031936.00133910 21406512

[B13] Mapstone J E D Roberts IG Strategies to improve recruitment to research studies John Wiley & Sons, Ltd 2007 2 17443634 10.1002/14651858.MR000013.pub3

[B14] Prout H B C Kinnersley P Robling M Hood K Tudor-Jones R A qualitative evaluation of implementing a randomized controlled trial in general practice Family Practice 2003 20 6 675 681 10.1093/fampra/cmg609 14701891

[B15] Campbell MK Recruitment to randomised trials: strategies for trial enrollment and participation study. The STEPS study Health technology assessment 2007 11 48 iii ix-105 17999843 10.3310/hta11480

