# Supplementary material for: Building an international network for a primary care research program: reflections on challenges and solutions in the set-up and delivery of a prospective observational study of acute cough in 13 European countries
Source: BMC Fam Pract. 2011 Jul 27;12:78. doi: 10.1186/1471-2296-12-78 (PMC3176157; doi:10.1186/1471-2296-12-78)
Supplement: Additional file 1 — Table 3: Challenges and solutions in tabulated format - this table details all the challenges and solutions observed in the GRACE-01 Study. [file 1471-2296-12-78-S1.PDF]

Additional File 1: Table detailing the challenges and solutions

Challenge	Sub domain<br>- Solution
**Selecting, setting up and maintaining PCNs**	**Selecting the right PCN (NNFs and NNCs)**<br>- Define clear selection criteria<br>- Use already established contacts<br>- Choose key people who have a prior interest in research area with at least one with a medical background<br>- Ensure key people have either prior experience in conducting studies or a willingness to learn<br>- Document roles and responsibilities given to key people involved<br>**Set up & maintaining the PCNs**<br>- Visit PCNs prior to recruitment to go through responsibilities, the study design, materials and the logistics of conducting the study<br>- Develop interactive training sessions<br>- Develop communication strategy and send to all involved – encourage constant communication across sites and with coordination team<br>- Build in "get to know each other" sessions part of group meetings<br>- Develop procedures and standardisation across PCN (SOPs, working practices)<br>- Set up group distribution lists for queries and to share important information<br>- Build in a period of reflection once patients recruitment is underway, to come together to share ideas and concerns and provide local solutions

Challenge	**Sub domain**<br><br>- Solution
**Designing local context-appropriate data collection tools and efficient data management systems**	**Culturally applicable data collection tools (CRF & Diary)**<br><br>-Involve key people (i.e. NNCs/NNFs) early in the development of study protocols and materials, ensuring they have an opportunity to contribute meaningfully to development.<br><br>- Hold face to face group meetings to discuss content<br><br>- Document differences in context, culture or healthcare systems<br><br>**High quality assurance**<br><br>- Translate all documents into local language and back translated into English<br><br>- Check back translations by one (if not two) members of the coordinating team, then check again - Make no assumptions<br><br>- Document all cultural or healthcare system differences<br><br>**High data management and quality**<br><br>- Develop central and local monitoring procedures<br><br>- Develop clear and effective data reporting processes<br><br>- Train PCNs in how to use the online system and provide instructions for use and document trouble shooting<br><br>- Central storage in encrypted database<br><br>- Run weekly real time monitoring reports to assess PNC efforts and reporting<br><br>- Provide an achievable target to enter data by (i.e. PCNs given a 2 day window in which to enter data)<br><br>**High recruitment rate, CRF and diary response rate**<br><br>- Hold face to face group meetings to openly share ideas<br><br>- Develop weekly newsletters comparing recruitment rates and response rates for PCNs and Clinicians<br><br>- Make reminder phone calls to participants<br><br>- Produce task lists to remind NNFs of important tasks.

Challenge	Sub domain<br><br>- Solution
**Gaining commitment and trust from all involved whilst maintaining enthusiasm**	**Communication and appreciation**<br><br>- Organise social events at meetings<br><br>- Give light-hearted prizes at annual dinners<br><br>- Take and distribute photos at all group meetings<br><br>- Send weekly bulletins on recruitment to all PCNs<br><br>- Continuously share ideas and group e-mails<br><br>-Produce GRACE certificates for Clinicians for CME certification purposes<br><br>-Produce weekly and quarterly newsletter to send to PCN/Clinicians<br><br>**Data ownership and publication strategy**<br><br>- Provide opportunity to become involved in contributing to GRACE-01 publications and to earn authorship of scientific publications<br><br>- Provide opportunity to suggest ideas for scientific publications both across country and within country<br><br>- Generate individual country reports<br><br>**Gaining commitment and trust**<br><br>- Establish a coherent philosophy (i.e. partners and collaborators became known as the “GRACE Family”)<br><br>- Involve key people (i.e. NNCs/NNFs) in the development of the study at a very early stage<br><br>- Conduct open meetings where opinions and thoughts can be voiced.
